# Osteitis fibrosa cystica—a forgotten radiological feature of primary hyperparathyroidism

**DOI:** 10.1007/s12020-017-1414-2

**Published:** 2017-09-12

**Authors:** Waldemar Misiorowski, Izabela Czajka-Oraniec, Magdalena Kochman, Wojciech Zgliczyński, John P. Bilezikian

**Affiliations:** 1 Endocrinology Dept, Medical Center for Postgraduate Education, Bielanski Hosp., Ceglowska 80 str., 01-809 Warsaw, Poland; 2 Department of Medicine, Endocrinology Division, College of Physicians and Surgeons, Columbia University, New York, NY USA

**Keywords:** Primary hyperparathyroidism, Osteitis fibrosa cystica, differential diagnosis

## Abstract

**Summary:**

Although bone disease and stone disease are the universally accepted classical manifestations of primary hyperparathyroidism, clinical parathyroid bone disease is rarely seen today in the United States (<5% of patients) and Western Europe. Nevertheless, in a given patient, classical skeletal involvement can be the first sign of primary hyperparathyroidism, but not recognized because it is not usually included, anymore, in the differential diagnosis of this manifestation of skeletal disease. We describe four cases of primary hyperparathyroidism in which the first clinical manifestation of the disease was a pathological fracture that masqueraded as a malignancy. The presence of large osteolytic lesions gave rise to the initial diagnosis of a primary or metastatic cancer. In none of the reported cases was primary hyperparathyroidism with *osteitis fibrosa* considered as the diagnosis. It would seem to us that this course is best explained by the fact that in many countries such manifestations of primary hyperparathyroidism have become a rarity. In fact, the incidence of *osteitis fibrosa* among patients with primary hyperparathyroidism in the US is estimated as so rare, that in majority of medical centers routine x-ray examinations of the bones in these patients is not recommended. The X-ray or computed tomography scan findings of *osteitis fibrosa cystica* include lytic or multilobular cystic changes. Multiple bony lesions representing brown tumors may be misdiagnosed on computed tomography scan as metastatic carcinoma, bone cysts, osteosarcoma, and especially giant-cell tumor. Distinguishing between primary hyperparathyroidism and malignancy is made readily by the concomitant measurement of parathyroid hormone which in primary hyperparathyroidism, again, will be markedly elevated. In the hypercalcemias of malignancy, such elevations of parathyroid hormone are virtually never seen.

**Conclusion:**

When radiographic evidence of a lytic lesion and hypercalcemia are present, primary hyperparathyroidism should always be considered in the differential diagnosis.

## Introduction

The clinical profile of primary hyperparathyroidism has changed markedly over the past several decades. Specific signs and symptoms of the disease previously featured skeletal disorders (*osteitis fibrosa cystica*, bone cysts, and brown tumors of the long bones), nephrolithiasis, and nephrocalcinosis. These overt complications are no longer evident in most patients, particularly in countries where biochemical screening for serum calcium is routine. In fact, overt skeletal disease in primary hyperparathyroidism is so infrequent in these countries that the classical feature of *osteitis fibrosa cystica* is seen rarely.

Nevertheless, in a given patient, classical skeletal involvement can be the first sign of primary hyperparathyroidism, but not recognized because it is not usually included, anymore, in the differential diagnosis of this manifestation of skeletal disease.

## Case 1

**Fig. 1:**
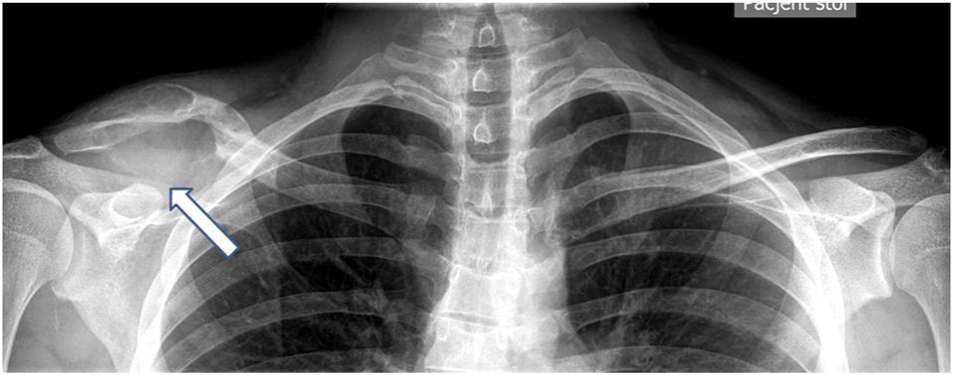
Case 1: x-ray of osteolytic tumor of the right clavicle (arrow)

**Fig. 2:**
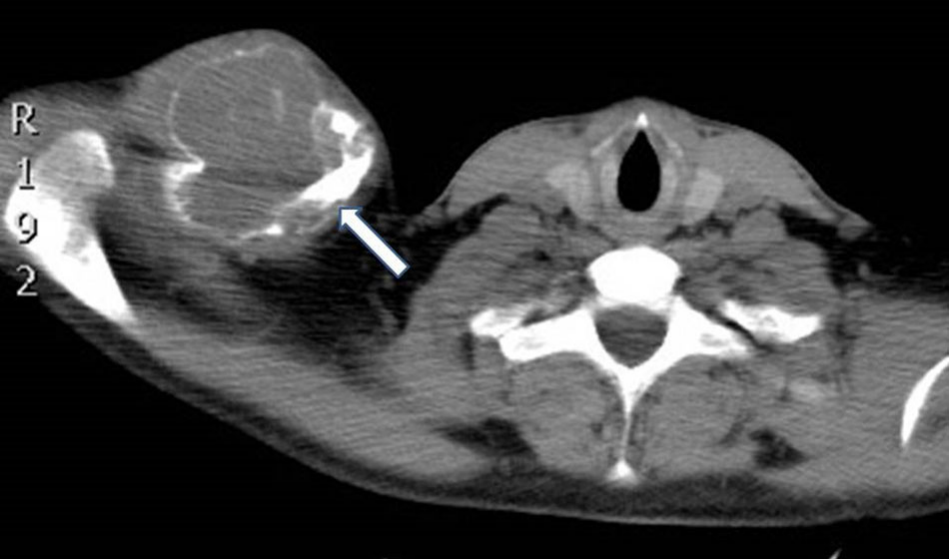
Case 1: CT scan of right clavicle tumor (arrow)

**Fig. 3:**
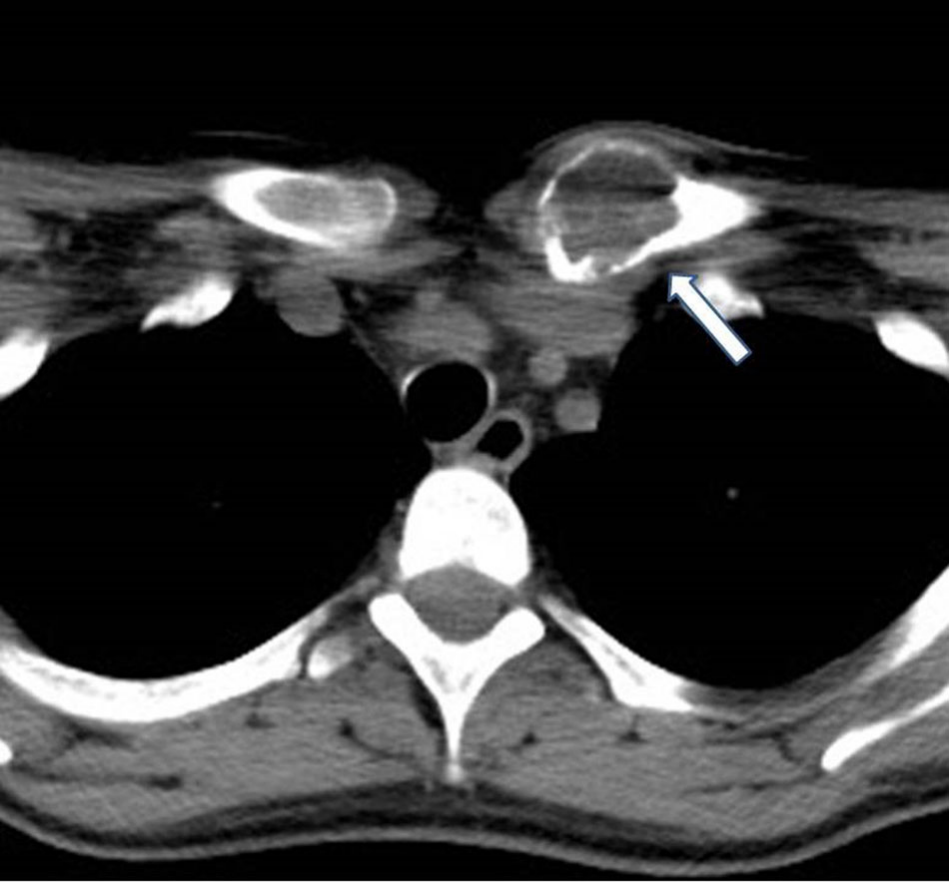
Case 1: CT scan—the similar osteolytic region of left clavicle (arrow)

A 28-year old man fractured his right clavicle without major trauma. X-ray showed a pathological fracture of the distal end of the clavicle, in the midst a large osteolytic bone tumor, without sharp borders (Fig. 1). The patient underwent bone biopsy of the lesion. After a cytological diagnosis of giant-cell tumor, was made, a second biopsy showed a brown tumor. Only then were biochemical indices obtained, confirming the diagnosis of primary hyperparathyroidism (PHPT): serum calcium was 13.2 mg/dL (nL, 8.4–10.2); serum phosphorus was 1.5 mg/dL (nL, 2.7–4.5). Albumin was 4.4 g/dL (nL, 3.9–4.8). Alkaline phosphatase activity was 624 IU/L (nL, 39–177), and parathyroid hormone (PTH) was 1560 pg/mL (nL, 10–65). Twenty-four-hour urinary calcium was 417 mg (nL, up to 250). Serum 25OHD was 12.0 ng/mL (nL, 20–20 ng mL) and 1.25(OH)_2_D was 96.3 pg/mL (nL, 25.0–86.5 spg/mL) (Figs. 2, 3).

## Case 2

**Fig. 4:**
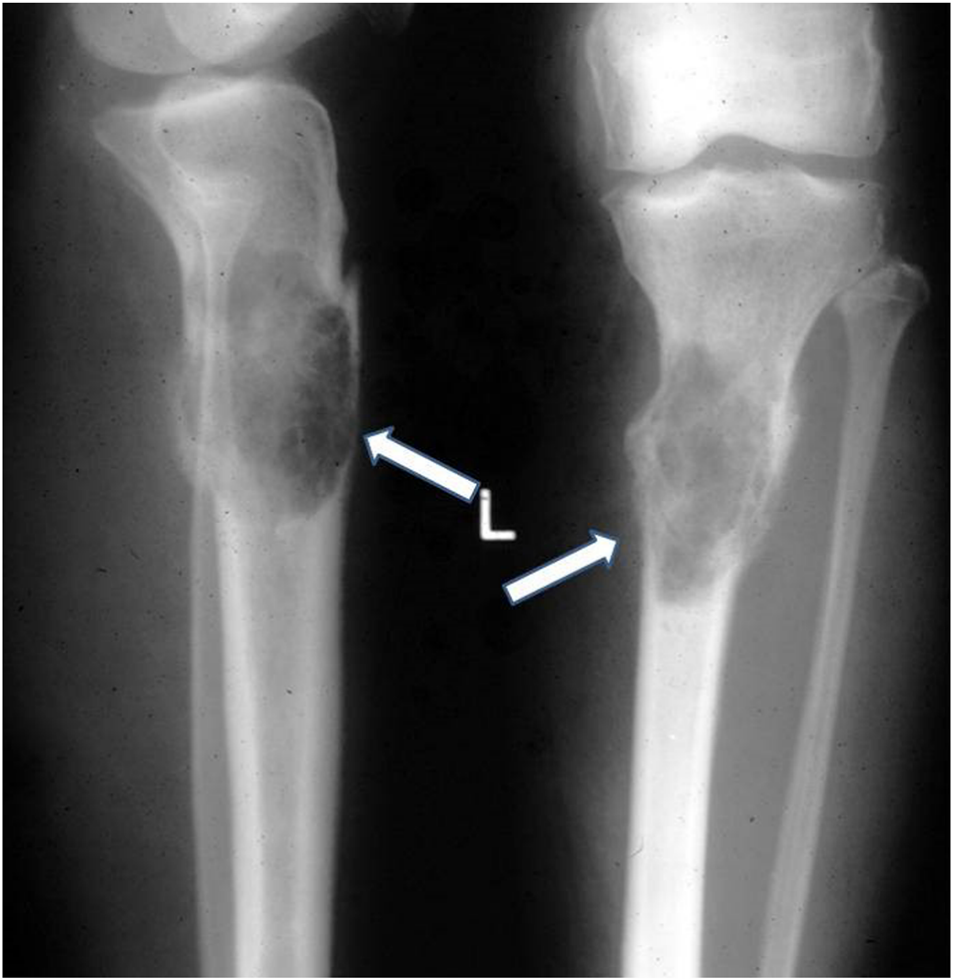
Case 2: x-ray: extensive osteolytic lesion in proximal end of tibia (arrows)

**Fig. 5:**
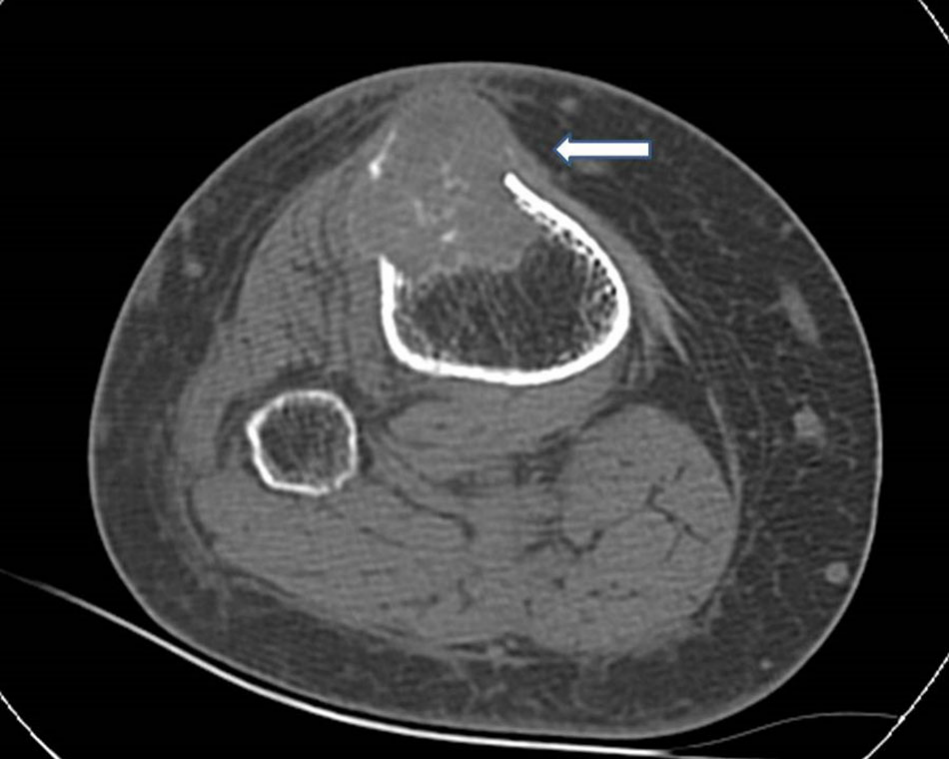
Case 3: CT scan of left proximal tibia: tumor-like extension of heterogenous soft tissue mass penetrating outside the bone (arrow)

**Fig. 6:**
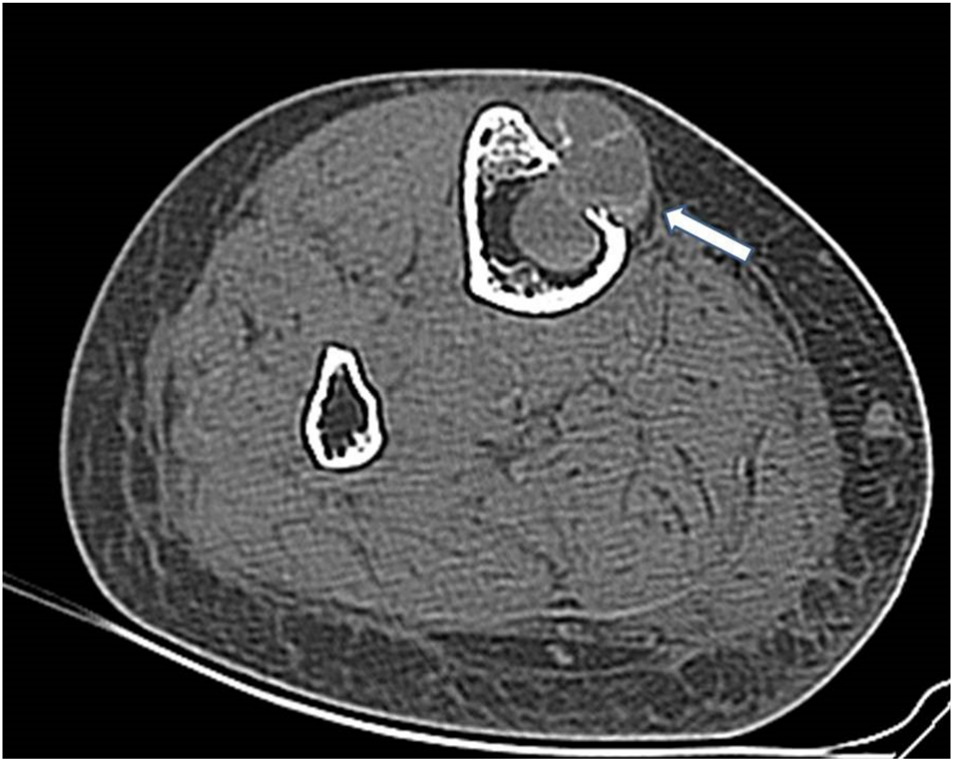
Case 3: CT scan of distal tibia: the similar lesion in distal end of the tibia

A 56-year old women sustained a left knee injury. X-ray revealed an extensive osteolytic lesion in the proximal tibia with cortical bone destruction along with a moth-eaten features (Fig. 4). Computed tomography (CT) scan showed a tumor-like extension of a heterogenous soft tissue mass extending beyond the bone boundaries (Fig. 5). Bone biopsy was consistent with osteogenic osteosarcoma and the patient was referred to an oncological center. Amputation was considered to be likely. Careful re-evaluation of biopsy indicated a brown tumor, rather than an osteosarcoma. Diagnosis was confirmed by elevated serum calcium and PTH: 13.8 mg/dL and 336 pg/mL, respectively. Serum phosphorus was 2.1 mg/dL, albumin was 4.1 g/dL and alkaline phosphatase activity was 356 U/L. Twenty-four-hour urinary calcium excretion was 380 mg. Serum 25OHD was 21.0 ng/mL (patient received cholecalciferol 2000 IU/d) (Fig. 6).

## Case 3

**Fig. 7:**
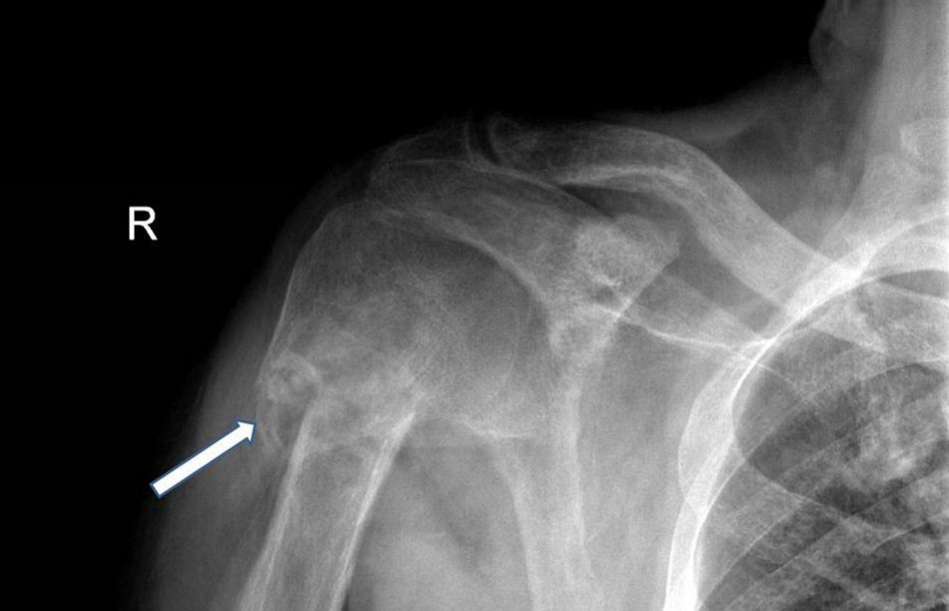
Case 3: x-ray of the pathological fracture, within extensive ostelytic lesion of right proximal humerus (arrow)

A 58-year old man who was under treatment for bilateral insertional Achilles tendinitis, sustained a fracture of the proximal right humerus. The impression was a pathological fracture, within an extensive osteolytic lesion. A deformed proximal end of the humerus was confirmed by X-ray and the patient was referred to the oncology service (Fig. 7). ^99m^Tc bone scintiscan revealed multiple focal lesions throughout the skeleton, particularly the spine, ribs, and pelvis. Over the next 2 months, hypercalcemia became evident. He was thought to have humoral hypercalcemia of malignancy (HHM). The patient received six cycles of palliative radiotherapy to the spine and pelvis. Eventually, a 2.0 cm tumor was discovered in the neck, posterior to the thyroid. Cytological transoesophageal biopsy was interpreted as “cancer cells”, and the patient was diagnosed as having “advanced thyroid cancer”. When he was referred to an experienced endocrinology surgeon, the possibility of PHPT was raised and confirmed. Serum calcium was 14.2 mg/dL, PTH 458 pg/mL, serum phosphorus 1.8 mg/dL and alkaline phosphatase activity was 459 U/L. Twenty-four-hour urinary calcium was 480 mg/24 h. Serum 25OHD and 1.25(OH)_2_D were 11.0 ng/mL and 88.5 pg/mL, respectively.

## Case 4

**Fig. 8:**
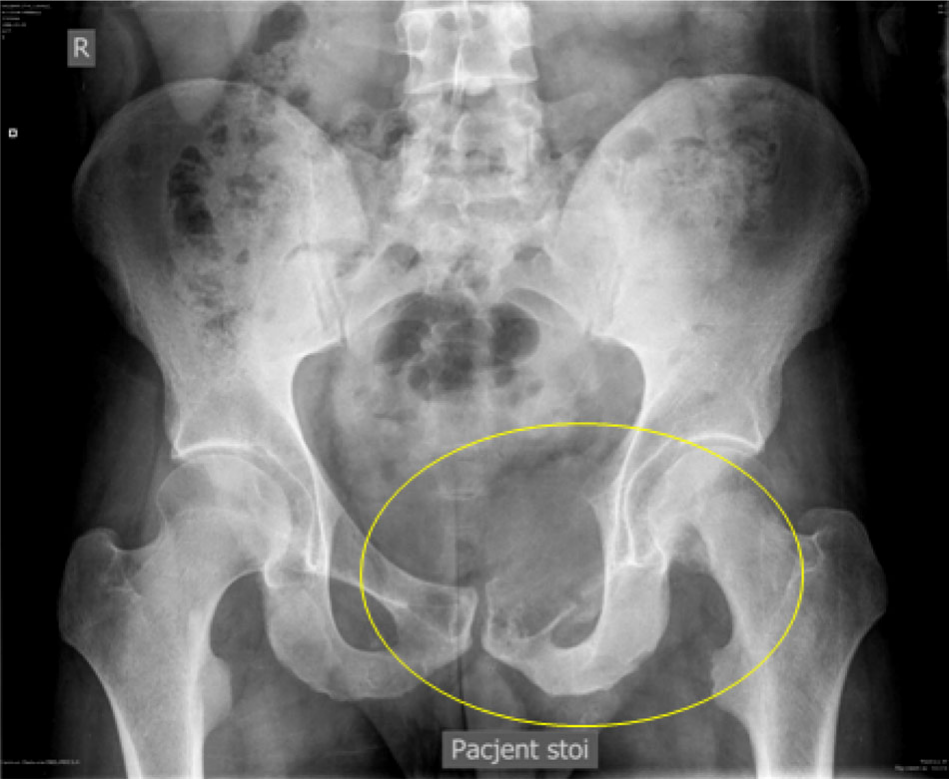
Case 4: x-ray of pelvis: large osteolytic lesion, deforming entire right pubic bone

**Fig. 9:**
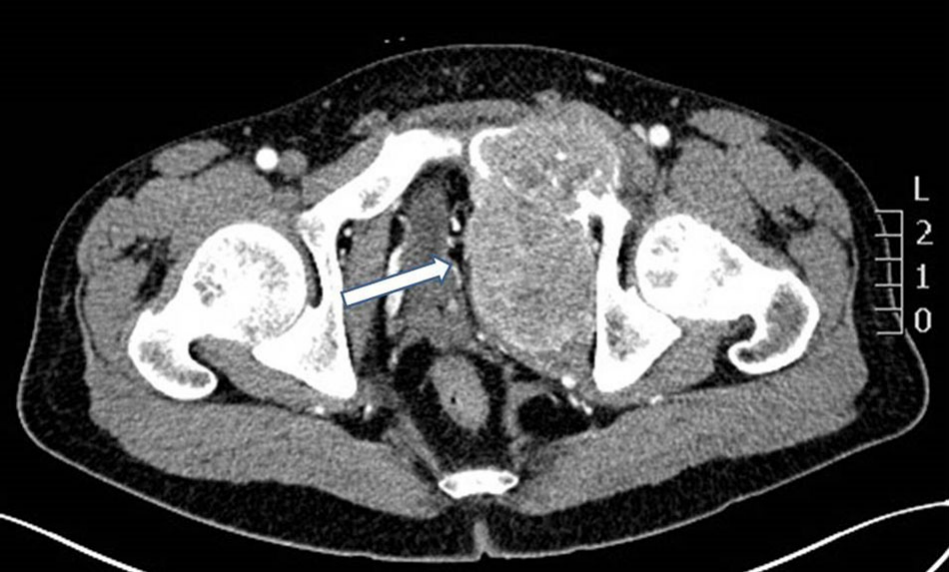
Case 4: heterogenic tumor, coming out of the pubic bone and filling nearly half of the small pelvis, compressing bladder (arrow)

A 23-year old man was referred to an orthopedic surgeon because of intensifying pelvic pain. A pathologic fracture within a large osteolytic lesion in a deformed right pubic bone was found by plain X-ray. CT scan revealed a very large tumor, originating from the pubic bone and occupying almost half of the pelvis, and compressing the bladder. Numerous similar osteolytic lesions were found throughout the skeleton. Thoracic X-ray and CT revealed an anterior mediastinum mass. A biopsy was performed via videothoracoscopy. On the basis of …“clear cells, probably metastatic”, the diagnosis of clear-cell renal cancer was made, despite the fact that arteriography of the kidney was normal. When his condition worsened, he received palliative chemotherapy. Worsening hypercalcemia was interpreted as HHM. After five courses of chemotherapy, patient was referred to National Cancer Institute, where the diagnosis of PHPT was evident: serum Ca was 14.6 mg/dL, PTH 1035 pg/mL, phosphorus 2.9 mg/dL, and urinary calcium was 210 mg/24 h. The estimated glomerular filtration rate was 55 mL/min/1.73 m^2^. Serum 25OHD and 1.25(OH)2D were 7.0 ng/mL and 96 pg/mL respectively. After Technetium (99mTc) sestamibi parathyroid scan/SPECT (three-dimensional) imaging parathyroid scintigraphy, a pathological mass in mediastinum was suggestive of ectopic parathyroid tissue which was eventually confirmed by surgery (Figs. 8 and 9).

## Commentary

We describe four cases of PHPT in which the first clinical manifestation of the disease was a pathological fracture that masqueraded as a malignancy. The presence of large osteolytic lesions gave rise to the initial diagnosis of a primary or metastatic cancer. In none of the reported cases was PHPT with osteitis fibrosa considered as the diagnosis. In the two cases in which symptomatic hypercalcemia was evident, the interpretation was that this was a manifestation of the malignancy itself. Even after bone biopsy was performed, the diagnosis was still thought to be a malignancy in two instances and palliative cancer treatment followed. In one case, leg amputation was considered and actually suggested to the patient. One might reasonably ask how could the “obvious” radiological appearance of osteitis fibrosa cystica be missed by the radiologists. It would seem to us that this course is best explained by the fact that in many countries such manifestations of PHPT have become a rarity and thus, not included in the differential diagnosis of such skeletal lesions.

The classical symptoms and signs of PHPT reflect the combined effects of increased PTH secretion and hypercalcemia. The classic abnormalities directly associated with hyperparathyroidism are stone and bone disease, both of which are typically due to long-standing exposure to PTH excess [1–3]. Although bone disease and stone disease are the universally accepted classical manifestations of PHPT, clinical parathyroid bone disease is rarely seen today in the United States (<5% of patients) and Western Europe. This classic manifestation of PHPT (*osteitis fibrosa cystica*), is characterized clinically by bone pain and radiographically by subperiosteal bone resorption, osteolysis of the distal clavicles, a “salt and pepper” appearance of the skull, bone cysts, and brown tumors of the bones. [1]. In a review of 97 cases of mild PHPT, for example, conventional radiography revealed signs of bone disease in only one patient [4]. In fact, the incidence of *osteitis fibrosa* among patients with PHPT in the US is estimated as so rare, that in majority of medical centers routine x-ray examinations of the bones in these patients is not recommended [5]. Whether overt bone disease reflects a delay in detecting PHPT in countries where routine biochemical screening is not practiced, or as seems equally plausible, is a manifestation of excess PTH action in the face of marginal or deficient vitamin D and calcium intake, remains to be determined. With regard to the use of routine biochemical screening, it has been shown that when such practices become more common, the clinical presentation of PHPT becomes less severe [6]. However, it is noteworthy that three of our four patients were markedly vitamin D deficient and the fourth one began vitamin D supplementation several months before diagnosis and. Many studies have now confirmed that manifestations of PHPT are worse when vitamin D deficiency is present. For example, the renal and skeletal manifestations of PHPT were much worse in a Chinese cohort, in which the average 25-hydroxyvitamin D concentration was 8.8 ng/mL, than in a United States cohort in which vitamin D deficiency was much less evident [7]. Even in mild PHPT, Walker et al. have shown that the biochemical and histomorphometric manifestations of primary hyperparathyroidism are worse [8]. Conversely, in centers where metabolic bone diseases are featured and evaluations tend to be much more extensive, another form of PHPT has been identified in which the total and ionized serum calcium concentration are normal [9]. The clinical presentations of PHPT, therefore, appear to vary according to a country’s disposition to routinely measure serum calcium, to be vitamin D deficient, and/or to be proactive in evaluation of metabolic bone diseases [10].

Brown tumor is a uni-focal or multi-focal bone lesion, which represents a terminal stage of hyperparathyroidism-dependent bone pathology [11]. This focal lesion is not a real neoplasm. In localized regions where bone loss is particularly rapid, hemorrhage, reparative granulation tissue, and active, vascular, proliferating fibrous tissue may replace the normal marrow contents, resulting in a brown tumor. The brown coloration is due to hemosiderin deposition. Brown tumors commonly affect the jaws, skull, pelvis, clavicle, ribs, femurs, and spine [12, 13]. They may cause swelling, pathological fracture, and bone pain. The X-ray or CT scan findings of *osteititis fibrosa cystica* include lytic or multilobular cystic changes [12]. Multiple bone lesions representing brown tumors may be misdiagnosed on CT scan as metastatic carcinoma, bone cysts, osteosarcoma and especially giant-cell tumor [14–18]. Because of the similarity of radiological features (e.g., cyst-like radiolucency) characteristic of other lesions, the diagnosis can be difficult. Scintigraphy is a highly sensitive method for the detection of the hyperparathyroidism-dependent bone pathology. However, it lacks specificity since a variety of diseases causing increased bone turnover such as trauma, infections, osteomalacia, and various metabolic bone diseases may be seen as increased foci of uptake. Metastases of cancer and multiple myeloma are also characterized by multiple focal lesions in similar locations on bone scintigraphy.

Brown tumors contain giant cells and spindle-shaped cells, intermixed with fibrous tissue and poorly mineralized woven bone [19, 20]. Histology cannot guarantee a certain diagnosis, as other lesions, such as giant cell tumor, giant cell granuloma, aneurysmal bone cyst, and some osteosarcoms show similar macroscopical and microscopical features, because all these conditions contain giant-cell lesions [21–25].

The most important way to distinguish these skeletal manifestations of advanced PHPT from malignancy is by biochemical analysis. When PHPT presents in this way, the serum calcium will always be elevated and, typically, to levels that are substantially above the upper limits of normal. Distinguishing between PHPT and malignancy is made readily by the concomitant measurement of PTH which in PHPT, again, will be markedly elevated. In the hypercalcemias of malignancy, such elevations of PTH are virtually never seen.

## Conclusion

When radiographic evidence of a lytic lesion and hypercalcemia are present, PHPT should always be considered in the differential diagnosis.
